# Gold Nanoparticle-Loaded Porous Poly(ethylene glycol) Nanosheets for Electrochemical Detection of H_2_O_2_

**DOI:** 10.3390/nano13243137

**Published:** 2023-12-14

**Authors:** Zhiyong Zhao, Michael Zharnikov

**Affiliations:** Angewandte Physikalische Chemie, Universität Heidelberg, Im Neuenheimer Feld 253, 69120 Heidelberg, Germany; wu182@uni-heidelberg.de

**Keywords:** hydrogen peroxide sensor, gold nanoparticles, poly(ethylene glycol), nanosheets, electrocatalysis

## Abstract

The effective detection of hydrogen peroxide (H_2_O_2_) in different environments and, above all, in biological media, is an important practical issue. To this end, we designed a novel electrochemical sensor for H_2_O_2_ detection by introducing gold nanoparticles (AuNPs) into the porous poly(ethylene glycol) (PEG) matrix formed by the thermally activated crosslinking of amino- and epoxy-decorated STAR-PEG precursors. The respective composite PEG-AuNP films could be readily prepared on oxidized Si substrates, separated from them as free-standing nanosheets, and transferred as H_2_O_2_ sensing elements onto the working electrode of the electrochemical cell, with the performance of the sensing element relied on the established catalytic activity of AuNPs with respect to H_2_O_2_ decomposition. The sensitivity, detection limit, and the operation range of the composite PEG-AuNP sensors were estimated at ~3.4 × 10^2^ μA mM^−1^ cm^−2^, 0.17 μM of H_2_O_2_, and 20 μM–3.5 mM of H_2_O_2_, respectively, which are well comparable with the best values for other types of H_2_O_2_ sensors reported recently in literature. The particular advantages of the composite PEG-AuNP sensors are commercial source materials, a simple fabrication procedure, the bioinert character of the PEG matrix, the 3D character of the AuNP assembly, and the possibility of transferring the nanosheet sensing element to any secondary substrate, including the glassy carbon electrode of the electrochemical cell. In particular, the bioinert character of the PEG matrix can be of importance for potential biological and biomedical applications of the designed sensing platform.

## 1. Introduction

Hydrogen peroxide, H_2_O_2_, is an important compound both in the industry and medicine. In particular, the production and elimination of H_2_O_2_ are closely correlated with the normal physiological processes in a human organism; thus, its concentration can serve as a marker for a variety of diseases, including cancer, with the respective cells featuring an enhanced concentration level of H_2_O_2_ compared to the normal tissue [1,2,3,4,5,6]. Also, the monitoring of H_2_O_2_ is important in plant science, and relies on oxidase-mediated H_2_O_2_ signaling pathways associated with the plant defense response [7]. Consequently, the effective detection of H_2_O_2_ in different environments, and above all in biological media, is an important practical issue. In this context, a variety of sensing platforms for H_2_O_2_ detection has been developed, relying on such detection mechanisms as surface plasmon resonance, fluorescence, luminescence, optical spectroscopy, colorimetry, titrimetry, and electrochemistry [6,8,9,10,11,12,13]. Among these techniques, electrochemical detection turned out to be particularly popular, employing both enzyme-based and non-enzymatic sensing platforms [14,15,16]. The enzyme-based detection involves the application of suitable enzymes that are capable of catalyzing H_2_O_2_ decomposition, such as horseradish peroxidase [17], hemoglobin, myoglobin [18,19], and cytochrome c [14]. However, the respective sensors frequently suffer from high cost, complicated design, and poor stability and reproducibility, with the latter drawbacks being related to the possible denaturation of enzymes and their sensitivity to environment conditions, such as pH and temperature [20,21]. These problems can be largely avoided in the case of non-enzymatic sensors, which are predominantly designed on the basis of noble metal nanoparticles (NPs), possessing distinct electrocatalytic properties with respect to H_2_O_2_ decomposition with the subsequent formation of H_2_O and O_2_ [22]. The most frequently used material in this context is gold [16,22], but platinum [23], silver [24] and alloy NPs [25,26] are effectively applied as well. In most cases, arrays of NPs are deposited or even directly grown on suitable supports, which then become a part of the electrochemical sensors. Recent representative examples include an array of hot-electron painted Au-Pt composite NPs grown on indium tin oxide without using reducing agents [26], electrodeposited silver NPs on the surface of two- and three-dimensional Zn-based metal-organic frameworks [24], an array of gold NPs (AuNPs) grown on porous carbon nanosheets [16], and an array of AuNPs on a cobalt-based and layered double hydroxide nanosheet [22]. The performance of the respective sensors varied to some extent depending on their architecture, with the most important parameters being the detection limit, sensitivity, selectivity, and composition range of H_2_O_2_. In most cases, amperometric sensing, involving measuring the current at a fixed potential that is suitable for the efficient reduction of H_2_O_2_, is used [16,22,24,26]. Within the respective electrochemical reaction, OH, created after the breakage of the O−O bond in H_2_O_2_, adsorbs on the surface of catalyst, leaves it as OH^−^ after the reduction, and, in the second reduction step involving one more OH group, transforms into O_2_ and H_2_O [22]. Alternatively, hydrogen ions can be involved, reacting with adsorbed OH radicals, gaining electrons from the active material, and forming H_2_O [13]. The respective reduction current is then recorded, with the value proportional to the H_2_O_2_ concentration. In most cases, a linear relation between this concentration and the recorded current is observed over a broad concentration range, serving as a calibration curve for the respective sensor [16,22,24,26]. The detection limit varies depending on the sensor design and is generally within the range of 100 nM–1 mM [13].

As observed by the above examples and in view of the importance of analyte diffusion for amperometric sensing [11], porous nanosheet supports are highly preferred. The porosity allows for the efficient assembly of NPs and their good accessibility by the target H_2_O_2_ during the sensing cycles. The nanosheet character allows for the off-site fabrication of the sensor, with its subsequent transfer into the electrochemical sensing device. A prospective material that meets these requirements are porous films built from so-called STAR-PEG (PEG–poly(ethylene glycol)) precursors, which can be prepared in a broad thickness range (10–300 nm) and have an additional advantage of being bioinert–favorable for their application in a biological environment [27,28]. Due to their distinct hydrogel character [27,29], these films can be loaded with NPs [27,30] and, both as unloaded and NP-loaded films, be separated from the primary substrate, forming free-standing nanosheets that are suitable for transfer onto any secondary substrate [30,31]. To this end, in the present study, we prepared composite PEG-AuNP nanosheets on a STAR-PEG basis and tested their performance for the electrochemical sensing of H_2_O_2_.

## 2. Materials and Methods

**Figure 1:**
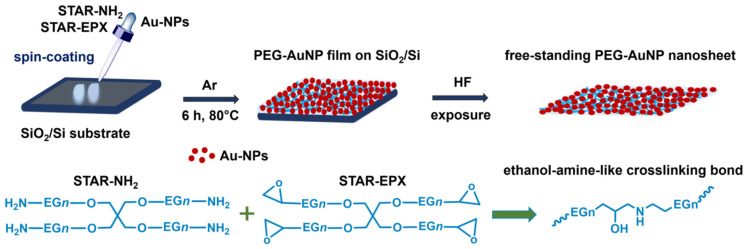
Schematic illustration of the fabrication procedure for the PEG-AuNP composite films and nanosheets, along with the chemical structures of STAR-NH_2_, STAR-EPX, and ethanol-amine-like crosslinking bonds. A monomer of the PEG arms of the precursors (−EG−) is described by the formula (−O−CH_2_−CH_2_−).

**Materials:** Hydrogen tetrachloroaurate(III) and trisodium citrate (99% purity) were purchased from Sigma–Aldrich and used for the synthesis of AuNPs. The amino-(NH_2_) and epoxy-(EPX) decorated, 4-arm STAR-PEG compounds (STAR-NH_2_ and STAR-EPX; Figure 1), with a molecular weight of 2000 g/mol (2k), were purchased from Creative PEGWorks (Chapel Hill, NC, USA). According to the specification of this company, these compounds are characterized by low polydispersity and high purity, viz. 99% for STAR-NH_2_ and 98% for STAR-EPX in terms of amine and epoxy substitution, respectively. According to the molecular weight, individual arms of these compounds comprise 9–11 EG monomers and have a length of 3.5–3.9 nm. SiO_2_-passivated Si(100) substrates were purchased from Siegert Wafer GmbH (Aachen, Germany). Other chemicals were purchased from Sigma-Aldrich (Taufkirchen, Germany) and used as received.

**Fabrication Procedure:** Citrate-stabilized AuNPs were synthesized according to the literature procedure [32,33], using hydrogen tetrachloroaurate(III) (99.9%) and trisodium citrate (99%) as the parent materials. A 1 mM solution (200 mL) of tetrachloroaurate(III) in Milli Q water (18 MΩ) was heated to boiling in a clean conical flask. Trisodium citrate (0.29 g) was dissolved in 15 mL of Milli Q water and added to the boiling HAuCl_4_ solution under stirring and reflux. Then, a color change in the above solution from yellow to burgundy over colorless (transparent), gray, and black was observed in 2 min. After 10 min, the reflux was stopped but the stirring continued for another 10 min. Finally, the solution was cooled and compensated with Milli Q water for the water loss during the reflux process. The average size of the NPs was estimated at ~15 nm (see below).

The PEG-AuNP composite films were prepared by a one-pot method, relying on the approach of ref. [34]. A trinary solution of the STAR-PEG precursors (either 10 or 30 mg/mL in water) and AuNPs (in water) was spin-coated onto the SiO_2_ passivated Si(100) substrates, and the PEG-AuNP composite films were directly obtained after a thermally promoted crosslinking process (6 h, 80 °C) under an argon atmosphere. The films were subsequently rinsed extensively with ethanol (99.8%) to remove the weakly bound material. Note that, alternatively, AuNPs could be loaded into the crosslinked PEG films by their immersion into AuNPs’ solution in water [27,30]. This, however, results in a gradient-like distribution of AuNPs in the films, with a higher concentration of the nanoparticles in the vicinity of the film-ambient interface [27,29]. In contrast, the one-pot preparation procedure should result in a higher loading and a homogeneous distribution of AuNPs in the PEG films. Note also that the standard solvent for the preparation of the PEG film is chloroform [27] but these films can also be prepared from toluene [34] and, as shown in the present study, from water.

As a reference to the PEG-AuNP composite films, pristine PEG film was prepared following the standard procedure [27]. Specifically, the 4-arm STAR-NH_2_ and 4-arm STAR-EPX were dissolved in chloroform and mixed in a 1:1 fashion, resulting in either a 10 mg/mL or 30 mg/mL precursor solution. This solution was then spin-coated onto the substrate and crosslinked by thermal annealing (6 h, 80 °C) under the argon atmosphere.

The separation of the reference PEG and composite PEG-AuNP films from the primary substrates and their transfer to the secondary substrates—either glassy carbon electrode (for the electrochemical experiments) or supporting Cu mesh (for characterization)—was performed according to the literature procedure [29]. Accordingly, the PEG films on the SiO_2_-passivated Si substrate were first exposed to HF to diminish their bonding to the substrate by the removal of the SiO_2_ overlayer and then separated from the substrate by an oblique immersion into water. The floating PEG-AuNP nanosheets were fished up from the surface of the water and transferred onto the secondary substrates.

**X-ray Photoelectron Spectroscopy:** The PEG-AuNP composite films were characterized by X-ray photoelectron spectroscopy (XPS). The measurements were performed with a MAX 200 (Leybold–Heraeus) spectrometer equipped with a Mg Kα X-ray source (260 W; ~1.5 cm distance to the samples) and a hemispherical analyzer (EA 200; Leybold–Heraeus). The spectra were collected in a normal emission geometry. The binding energy (BE) scale of the spectra was referenced to the Au 4f_7/2_ peak of gold at 84.0 eV [35]. The measurements were carried out at room temperature and under ultra-high vacuum conditions (a base pressure of 3 × 10^−9^ mbar).

**Scanning Electron Microscopy:** The PEG-AuNP composite films and nanosheets were characterized by the scanning electron microscopy (SEM). The measurements were performed with a LEO 1530 scanning electron microscope (Zeiss, Oberkochen, Germany). The acceleration voltage was set to 3 keV.

**Electrochemical Experiments:** The electrochemical experiments were carried out with a Zahner potentiostat (model IM6E). All measurements, encompassing cyclic voltammetry (CV) and amperometric *i*-*t* (current-time) testing, were performed within a customized three-electrode electrochemical cell in which a KCl-saturated Ag/AgCl electrode and a platinum electrode were used as the reference and counter electrodes, respectively. In addition, glassy carbon electrode (GCE) with a diameter of 3 mm, surface-modified with either a PEG or PEG-AuNP nanosheet, was used as the working electrode. All the electrodes were purchased from Ossila (Sheffield, UK). The electrolyte utilized in all the experiments consisted of a phosphate buffer solution (PBS, pH = 7), containing varying concentrations of H_2_O_2_.

CV measurements were conducted within a potential window of −0.5 V to +0.5 V (vs. Ag/AgCl). Different scan rates, ranging from 10 to 500 mV/s, were applied. The recording of the amperometric *i*-*t* curves was carried out under a constant potential of −0.2 V (vs. Ag/AgCl); specific quantities of H_2_O_2_ were then successively added into the PBS electrolyte.

## 3. Results and Discussion

### 3.1. Characterization by XPS

**Figure 2:**
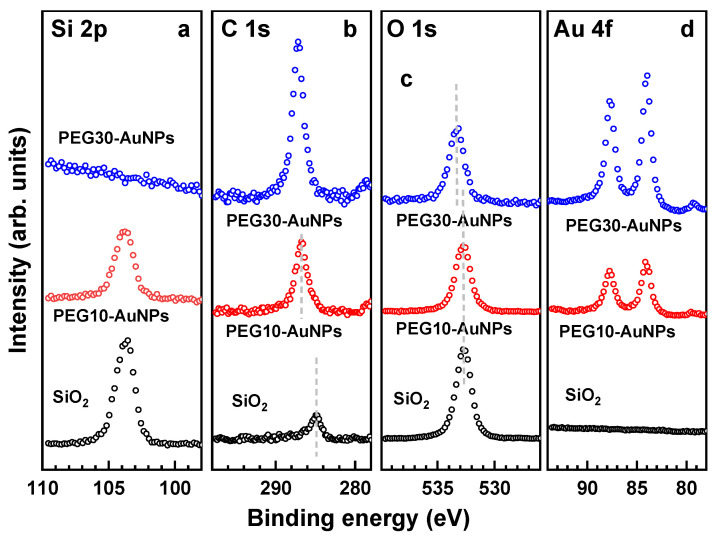
Si 2p (**a**), C 1s (**b**), O 1s (**c**) and Au 4f (**d**) XP spectra of SiO_2_ passivated Si(100) substrate and the PEG-AuNP films on this substrate, prepared at a concentration of the 4-arm STAR-NH_2_ and 4-arm STAR-EPX precursors in the primary solution of either 10 or 30 mg/mL. The positions of some peaks are traced by the vertical light gray dashed lines.

XP spectra of the SiO_2_ passivated Si(100) substrate and PEG-AuNP films on this substrate, prepared at a concentration of the 4-arm STAR-NH_2_ and 4-arm STAR-EPX precursors in the primary solution of either 10 or 30 mg/mL, are shown in Figure 2. These films are denoted as PEG10-AuNPs and PEG30-AuNPs, respectively. Because of the thick SiO_2_ layer of the substrate, a partial charging of all the spectra occurred, which was corrected for by their shift by −0.45 eV.

The spectra of the substrate exhibit the characteristic signals of SiO_2_ at 103.7 eV for Si 2p and 532.7 eV for O 1s, in good agreement with the reference literature values (103.6 eV and 532.5–533.0 eV, respectively) [35]. The surface of the substrate is slightly contaminated, as shown by the presence of a small C 1s signal at ~284.9 eV.

The spectra of the PEG10-AuNP film exhibit the characteristic [27] C 1s signal of the PEG films at 286.7 eV and the Au 4f signal (Au 4f_7/2, 5/2_ doublet) of the AuNPs at 84.0 eV (Au 4f_7/2_), but the O 1s signal at 532.7 eV is dominated by the contribution of the substrate (the same BE). The film is obviously very thin, which is supported by the rather strong Si 2p signal, exhibiting, as compared to the bare substrate, only weak attenuation. Presumably, at this concentration of the STAR-PEG precursors, a thick and homogeneous film is not formed, which can be both related to the specific solvent (water) and the presence of the AuNPs in the primary trinary solution. However, the situation changes dramatically in the PEG30-AuNP case. The Si 2p signal of the substrate becomes not visible anymore, suggesting its strong attenuation by the thick PEG30-AuNP overlayer, the C 1s and Au 4f signals increased strongly in intensity, and the O 1s signal appears at a higher binding energy (~533.3 eV) compared to that of the substrate (532.7 eV), now representing the PEG film. Thus, the PEG30-AuNP film is rather thick, in accordance with the expectations (~130 nm) [29]. Moreover, the intensity relations between the PEG10-AuNP and PEG30-AuNP films do not necessarily reflect the entire difference in the thickness of these films and the extent of the AuNP loading, since the intensities of the C 1s, O 1s, and Au 4f features for the PEG30-AuNP film are most likely limited by self-attenuation. It is well known that the intensity of an XPS signal from a film with a thickness *d* is defined by the term (1 − exp(−*d*/*λ*)), where *λ* is the attenuation length of the photoelectrons at the given kinetic energy [36,37]. Consequently, if *d* >> *λ*, the signal does not scale any more with *d* (self-attenuation) but becomes independent of it. This situation occurs indeed for the PEG30-AuNP film, the thickness of which (~130 nm) [29] significantly exceeds the relevant *λ* values (3.3–5.0 nm) [27].

Based on the above data, we concluded that the comparably thin PEG10-AuNP films are most likely not sufficiently robust to survive the separation from the primary substrate and transfer to the secondary support. In contrast, the PEG30-AuNP films, featuring a high AuNP loading, should most likely fulfil all the requirements. Consequently, only such films, denoted for the sake of brevity to just PEG-AuNP, below, were used for all subsequent experiments.

### 3.2. Characterization by SEM

**Figure 3:**
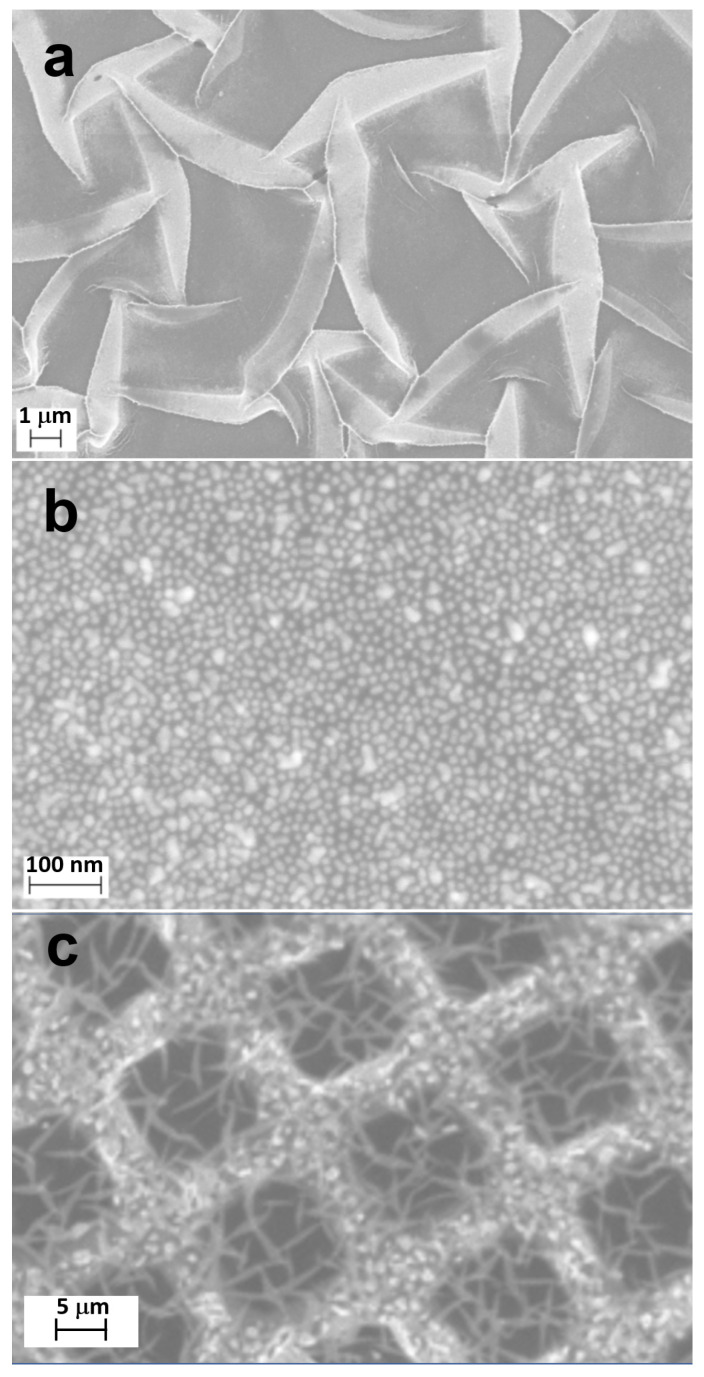
(**a**) Large-scale SEM image of a PEG-AuNP film; (**b**) high-resolution SEM image of this film; and (**c**) an image of this film after its transfer onto supporting, quadratic Cu mesh. The gray and nearly white areas in (**a**) represent unfolded parts of the nanosheet and folds, respectively.

Representative SEM images of the PEG-AuNP films and nanosheets are shown in Figure 3. A large-scale image in Figure 3a shows a homogeneous film, which, however, has some folds. We think that the folds are a consequence of using water as the solvent, which was necessary to combine the 4-arm STAR-NH_2_ and 4-arm STAR-EPX solutions with the AuNP solution within the one-pot procedure. Note that no folds are usually formed at the preparation of the PEG film from chloroform, which is the standard solvent for the STAR-PEG precursors [27,29].

A high-resolution SEM image of a PEG-AuNP film in Figure 3b exhibits a homogeneous distribution of AuNPs with an average diameter of ~15 nm. The extent of agglomeration is very low; it cannot be excluded that the respective, larger spots originate from an overlap of spots from several individual NPs located at the different vertical positions in the film with respect to the substrate. Note that the thickness of the PEG-AuNPs’ film at the given concentration of the 4-arm STAR-NH_2_ and 4-arm STAR-EPX precursors is expected to be close to ~130 nm [29], which is larger than the average size of the AuNPs, so that the signals of several NPs in the vertical projection of the SEM image can overlap. Note also that the average size of the AuNPs is higher than the average mesh size of the PEG network, which, according to the arm length of the precursors (see Section 2), is ~8 nm. Thus, AuNPs are physically embedded into this network, most likely without having any specific chemical links to the PEG chains.

The image of a PEG-AuNP film, transferred as a free-standing nanosheet onto the supporting Cu mesh in Figure 3c, shows that these films are sufficiently robust and capable of transfer to any secondary substrate, including GCEs (electrochemical experiments), in particular. The folds (Figure 3a), conversed upon the transfer (nearly gray areas in the mesh openings in Figure 3c), are obviously not a problem.

### 3.3. Electrochemistry

**Figure 4:**
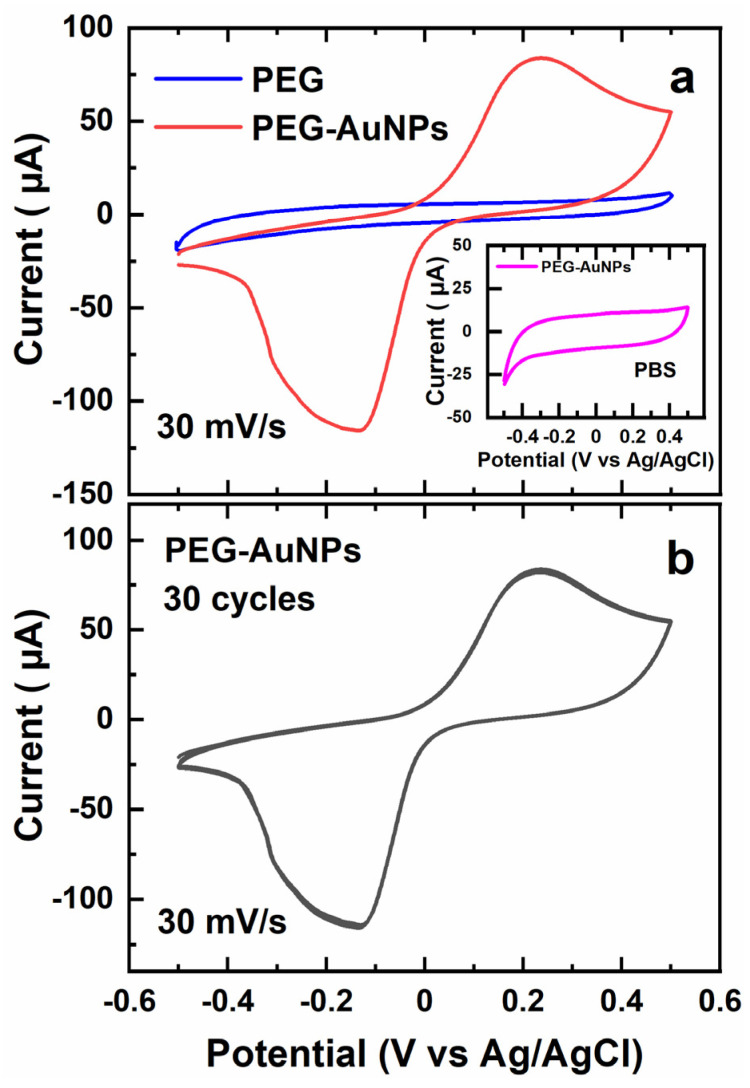
(**a**) CV curves for the reference, pristine PEG film (blue line) and the composite PEG-AuNP film (red line); (**b**) 30 subsequent CV curves for the composite PEG-AuNPs’ film; the individual curves overlap strongly and cannot be distinguished. The measurements were performed in PBS at the presence of 2 mM H_2_O_2_. Inset: CV curve for the composite PEG-AuNP film at the presence of PBS only. The scan rate was set to 30 mV/s for all CV curves shown.

CV curves for the reference, pristine PEG film (30 mg/mL precursors solution) and the composite PEG-AuNP film are presented in Figure 4a. Whereas the curve for the pristine film does not exhibit any redox features, that for the composite film shows a pronounced reduction peak at ca. −0.15 V, which is associated generally with the electrocatalytic decomposition of H_2_O_2_ by noble metal NPs [15,17], which, in our case, were mediated by the AuNPs embedded into the porous PEG network. Also, the reference CV curve, measured for the composite PEG-AuNP film in the presence of PBS only (inset of Figure 4a), does not exhibit any redox features, and serves as additional evidence for the role of H_2_O_2_.

The repetition of CV scans does not result in any visible change of CV curves at the presence of H_2_O_2_ (Figure 4b), which suggests stable conditions for the electrocatalytic reaction and a high stability of the composite catalyst.

**Figure 5:**
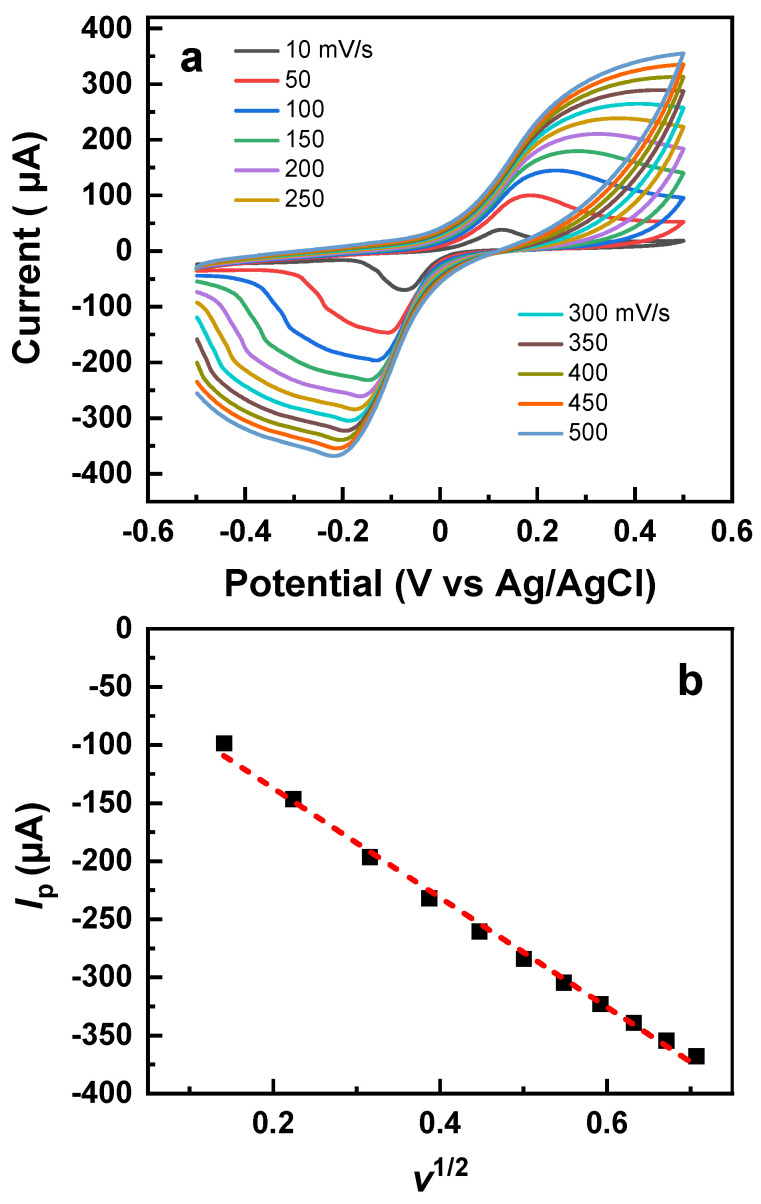
(**a**) CV curves for the composite PEG-AuNP film acquired at different scan rates; (**b**) plot of the anodic peak current, *I_p_* vs. *v*^1/2^ (black-filled squares) along with a linear fit of the experimental data (red dashed line).

The exact position of the redox peaks and the values of the anodic and cathodic peak currents depend on the scan rate, as shown in Figure 5a, where the CV curves for the composite PEG-AuNP film for a variable scan rate (10–500 mV/s) are presented. With the increasing scan rate, the position of the reduction peak shifts progressively to the lower potentials while the respective peak current increases. The increase in the peak current, *I_p_*, can be generally described by the Randles–Sevcik equation

*I_p_* = *nFAD*^1/2^*C*_0_*v*^1/2^ (1)

where *n* is the number of electrons transferred in the redox event, *F*—Faraday constant, *A*—electrode area, *D*—diffusion coefficient, *C*_0_—concentration, and *v*—the scan rate [38]. Indeed, a plot of *I_p_* vs. *v*^1/2^ in Figure 5b provides a nearly linear relation between these parameters, which suggests that the reduction process in the given case is adequately described by the Randles–Sevcik equation. This means that this process has a diffusion-controlled and reversible character, with no major structural changes in the analyte—the composite PEG-AuNP film.

**Figure 6:**
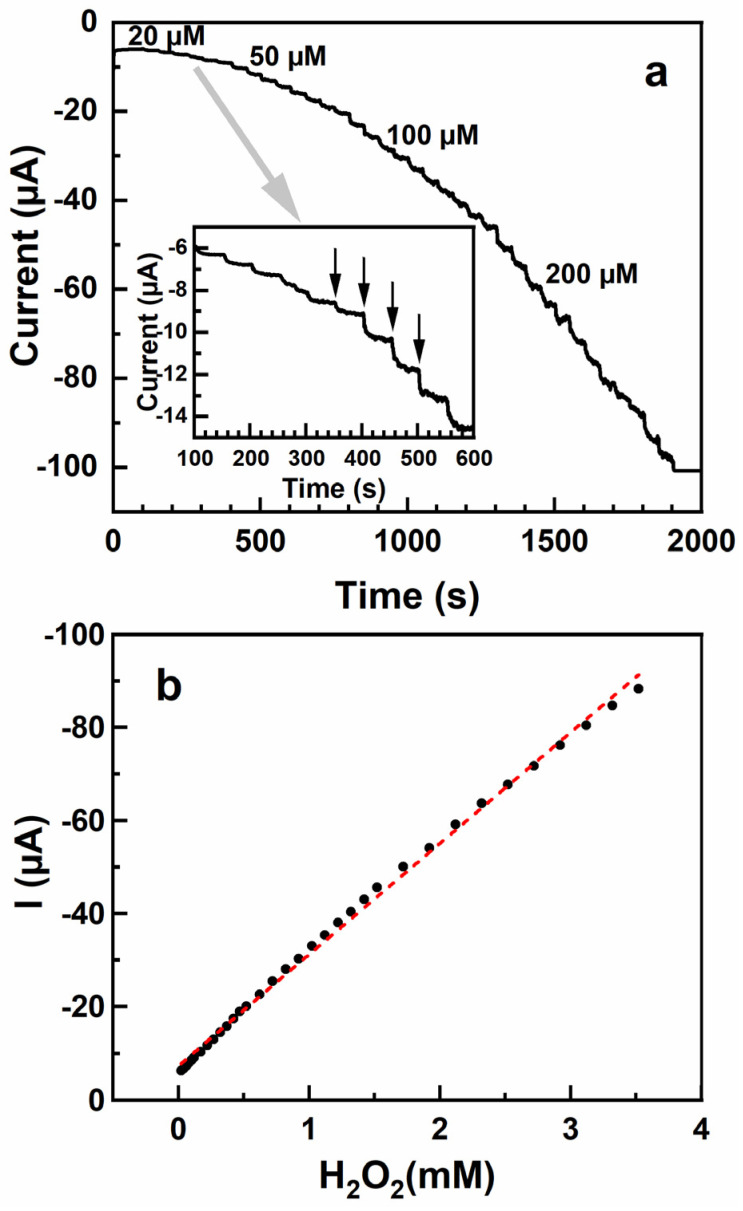
(**a**) Amperometric response of the composite PEG-AuNP film to increased H_2_O_2_ concentration upon successive, stepwise addition of 20 μM (100–350 s), 50 μM (400–750 s), 100 μM (800–1250 s), and 200 μM (1300–1900 s) of H_2_O_2_ to 50 mL of PBS buffer. Inset: zoomed presentation of the response to the smallest doses of H_2_O_2_. Several successive points in time of H_2_O_2_ addition are exemplarily shown by vertical arrows. The potential was set to −0.2 V. (**b**) Current response to the H_2_O_2_ concentration (black-filled circles), along with a linear fit to the experimental data (red dashed line).

Based on the CV curves in Figure 5a, −0.2 V was selected as the operation potential for the amperometric response experiments, termed frequently as *i*-*t* tests [26,39]. Within these experiments, different amounts of H_2_O_2_ were stepwise added into the electrolyte and the resulting anodic peak current was recorded. The respective steady-state vs. time curve is shown in Figure 6a. Accordingly, the current increases successively, in a stepwise fashion, upon the addition of H_2_O_2_ to the electrolyte. The value of the current step depends, as expected, on the amount of the added H_2_O_2_, which is particularly well visualized in the inset of Figure 6a, where the amperometric response of the composite PEG-AuNP film to the smallest amounts of H_2_O_2_ (20 and 50 μM) is shown. Note that the reference value of the current, for the background electrolyte only (time = 0 s), is very small (~6 μA).

The resulting relation between the recorded current and the H_2_O_2_ concentration is shown in Figure 6b. This relation can be adequately fitted by a straight line (R^2^ = 0.997; according to the fit parameters) over the entire concentration range, even though small systematic deviations from the exact linearly behavior cannot be denied. According to the fitting curve, the response sensitivity of the composite PEG-AuNP film was estimated at ~24 μA mM^−1^ or, considering the GCE area (0.071 cm^2^), 3.4 × 10^2^ μA mM^−1^ cm^−2^. The detection limit was determined via the standard sensitivity criterium applied to analogous sensors [16,24,26]. viz. the 3 S_b_/S method, where 3 represents the signal-to-noise ratio, S_b_ is the standard deviation of the current response in the absence of H_2_O_2_, and S is the slope of the calibration curve (Figure 6b). According to this method, the detection limit was estimated at 0.17 µM of H_2_O_2_. The latter value is well comparable with the parameters of the analogous, differently designed H_2_O_2_ sensors reported recently in literature, including an array of hot-electron painted Au-PtNPs on indium tin oxide (0.11 μM) [26], AgNP-modified two-dimensional Zn-based metal-organic framework (1.67 μM) [24], porous carbon nanosheet-supported AuNPs (1.044 μM) [16], cobalt-based and layered double hydroxide nanosheet-supported AuNPs (0.19 mM) [22], and carbon microfibers coated with nafion and silver NP composite (0.48 μM) [40]. Also, a recent review on the H_2_O_2_ detection provides the detection range of 100 nM–1 mM for the H_2_O_2_ sensors [13], so that we are close to the low limit of this range. The response sensitivity and the detection range are comparable as well, even though these parameters vary to some extent from study to study, depending on the specific sensor design and the range of the concentrations tested.

Along with the sensitivity, detection range, and detection limit discussed above, the selectivity of the composite PEG-AuNP sensor was verified, mimicking the literature approach [16,22,24,26]. For this purpose, this sensor was exposed to a variety of potential interfering compounds and biomolecules, frequently found in biological samples, such as ascorbic acid (L-AA), biotin, and fibrinogen from human plasma. The first two compounds are electroactive ones at the presence of AuNPs [41,42], whereas fibrinogen is not electroactive but, as a protein, is potentially capable of adsorbing on the surface of AuNPs [43], diminishing their activity with respect to H_2_O_2_, and changing, thus, the amperometric signal.

**Figure 7:**
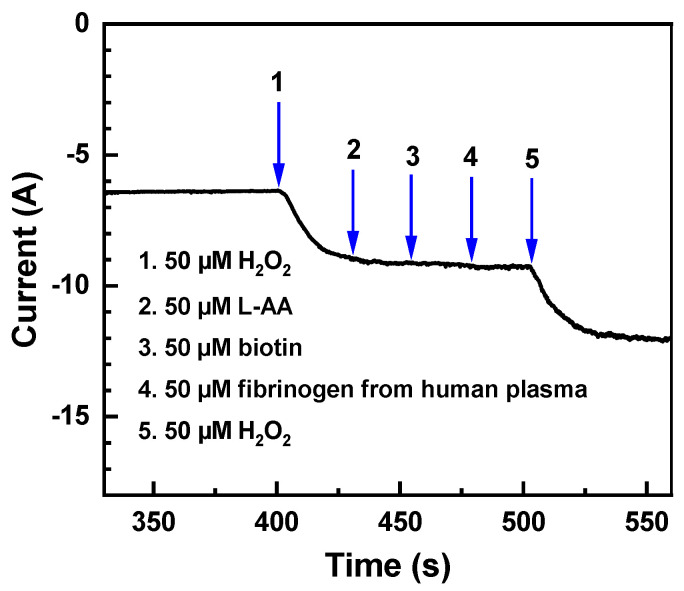
Amperometric response of the composite PEG-AuNP film to the stepwise injection of H_2_O_2_ and potentially interfering substances into 50 mL of PBS. The potential was set to −0.2 V, which, according to the CV data (Figure 5), was the optimal value for the H_2_O_2_ sensing by amperometry.

These compounds were successively added to the electrolyte, resulting in no noticeable current response at the given, preselected potential, as shown in Figure 7. In contrast, the addition of the same amount of H_2_O_2_ before and after the anti-interference tests resulted, as expected, in the pronounced increase in the recorded current (Figure 7), proving the readability and active character of the sensor. Significantly, the changes of the current in steps 1 and 5 are exactly the same (see Figure 7), which proves that there is no diminishing of the PEG-AuNP sensor activity associated with the adsorption of fibrinogen on the surface of AuNPs. Note that, along with this finding and the lack of the electrochemical activity at the given potential for ascorbic acid and biotin, it is also of importance that all potentially interfering compounds were not captured in the sensor matrix, changing its electric and permeability parameters. In this present case, this was a direct consequence of the distinct bioinert character of the PEG matrix, representing a particular advantage of the PEG-AuNP composite sensor, which is important for H_2_O_2_ sensing in biological environment and medical samples.

## 4. Conclusions

We have designed and tested a novel H_2_O_2_ sensor on the basis of the PEG-AuNP composite nanosheets. The citrate-stabilized AuNPs with diameters of ~15 nm were synthesized and introduced into the porous PEG matrix by a one-pot crosslinking reaction with the PEG-NH_2_ and PEF-EPX precursors. The AuNPs were then captured physically in the PEG matrix in a 3D fashion, being, at the same time, well accessible for H_2_O_2_ molecules because of the matrix porosity, which was also advantageous for its high permeability for electrochemical species and ions within the electrochemical cell. In spite of the citrate stabilization, AuNPs were well capable of electrochemical catalyzing the decomposition of H_2_O_2_, as emphasized by the appearance of the respective reduction peak in the CV curves (Figure 4). The corresponding electrocatalytic reaction represents the basic process for the electrochemical sensing of this important compound. The detection limit of the composite PEG-AuNP sensor was estimated at 0.17 μM of H_2_O_2_, which is well comparable to the best values for analogous sensors, as reported recently in the literature. The sensitivity of the PEG-AuNP sensor was estimated at ~3.4 × 10^2^ μA mM^−1^ cm^−2^ over the detection range of 20 μM–3.5 mM, with a close-to-linear relation between the H_2_O_2_ concentration and the recorded current. The selectivity of the sensor with respect to H_2_O_2_ was demonstrated by the control’s anti-interference experiments with several potentially interfering compounds.

Apart from the above favorable parameters, the advantages of the composite PEG-AuNP sensor are the commercial source materials; simple fabrication procedure; bioinert character of the PEG matrix; 3D character of the AuNP assembly; and the possibility of transferring this nanosheet sensor to any secondary substrate, including the GCE electrode of the electrochemical cell, in particular. Potentially, the sensor can be further optimized, e.g., by changing the size and composition of the nanoparticles, which can result in its even better performance. Along with custom-synthesized NPs, commercial NPs, that are both standard and tailor-made and available on the market, can be used, simplifying even further PEG-NP fabrication procedure. In particular, the bioinert character of the PEG matrix can be of importance for potential biological and biomedical applications of the designed PEG-NP sensing platform.

## Data Availability

The data are available on request from the corresponding author.
